# Development and Worsening of Hypertension with Age in Male Wistar Rats as a Physiological Model of Age-Related Hypertension: Correction of Hypertension with Taxifolin

**DOI:** 10.3390/ijms252011216

**Published:** 2024-10-18

**Authors:** Elena A. Tukhovskaya, Alina M. Ismailova, Natalya A. Perepechenova, Gulsara A. Slashcheva, Victor A. Palikov, Yulia A. Palikova, Dmitry I. Rzhevsky, Vladimir A. Rykov, Nadezhda I. Novikova, Igor A. Dyachenko, Arkady N. Murashev

**Affiliations:** Biological Testing Laboratory, Shemyakin-Ovchinnicov Institute of Bioorganic Chemistry (Branch), Russian Academy of Sciences, Prospekt Nauki, 6, Pushchino 142290, Russia

**Keywords:** hypertension, Wistar rat, age, animal models, taxifolin

## Abstract

To preclinically study the effectiveness of new antihypertensive drugs, various animal hypertension models are used. However, most of them do not correspond to primary hypertension, which develops in people with age. We used male Wistar rats of 4, 10, 12 and 18 months old. The animals were divided according to systolic blood pressure (SBP) into normotensive (SBP ≤ 114 mmHg) or hypertensive (SBP ≥ 115 mmHg). Within hypertensive animals, two cohorts were distinguished—with SBP below and above 125 mmHg. The animals received 100 µg/kg of taxifolin intraperitoneally for 7 days. A significant difference was shown between animals with SBP above and below 115 mmHg, as well as between cohorts of hypertensive animals with SBP above and below 125 mmHg within each age. The number of animals with elevated SBP increased with age both for clusters with an SBP above 115 mmHg and for cohorts with an SBP above 125 mmHg. Administration of taxifolin led to a significant decrease in the SBP only in hypertensive animals. A physiological model of age-related hypertension was obtained in male Wistar rats. It has been shown that hypertension develops and worsens with age. In preclinical studies, it should be taken into account that drugs may have different effects depending on the initial SBP of the animals.

## 1. Introduction

Hypertension affects 1.28 billion adults aged 30–79 years and is a leading cause of death worldwide [1] due to the fact that it represents a major risk factor for the development of cardiovascular diseases (CVD), such as stroke and myocardial infarction [2,3,4]. Hypertension affects over 30% of the world’s adult population [5]. Among older people, the proportion of hypertensive patients is more than 60% [6]. It has been established that the annual mortality rate associated with high blood pressure is 7.7–10.4 million people worldwide [7]. A study by Ly et al. 2019, which measured out-of-office blood pressure, calculated the risks of developing fatal and non-fatal CVDs, such as heart attacks and strokes, in people based on age when their SBP increased by 20 mmHg. This study showed that the relative risk (per 20 mmHg increase in the SBP) of developing CVD events is higher in young people, while the absolute risk (per 1000 person-years) of developing CVD events is significantly higher in older people [8]. It has been shown that at the age of 40–69 years, each 20 mmHg difference from the normal SBP is associated with a more than twofold increase in stroke mortality [9]. Another study showed an increased risk of mortality from early-onset CVD [10]. However, the increase in the elderly population is bringing this group suffering from hypertension to the forefront of healthcare efforts aimed at treating hypertension [11]. According to the American Heart Association, systolic blood pressure can be classified as follows: < 120 mmHg—normal, 120–129 mmHg—elevated, 130–139 mmHg—stage 1 hypertension, ≥ 140 mmHg—stage 2 hypertension [12,13]. With age (when comparing subgroups under 45 years of age versus those over 65 years of age), the risk of developing hypertension increases by an order of magnitude equally in men and women [14]. Although (as of 2016) there is a decrease in the prevalence of age-related hypertension among women compared to men, but only in high-income countries [6]. The mechanism of development of hypertension is closely related to the processes that occur during the aging of the body, the so-called “Vascular Health Triad”. This was discovered to be related to aging [15,16], and hypertension [17], involving a combination of pathological factors such as chronic inflammation, oxidative stress and vascular dysfunction. These three factors are interrelated and mutually influence the formation of hypertension. Thus, an increase in the formation of free radicals during inflammation leads to the launch of the NO cascade, which activates prostaglandins and leads to oxidative post-translational modification of proteins affecting vascular and cellular signaling pathways [17]. The resulting vascular dysfunction leads to vascular resistance and, as a consequence, an increase in blood pressure [18]. Vascular dysfunction, in turn, contributes to the further development of inflammation and oxidative stress [15,16,17]. At the same time, the vascular endothelium, which normally produces NO as a protective mechanism of vasodilation and inhibition of leukocyte adhesion [19], with aging and hypertension, produces vasoactive molecules such as endothelin-1 (ET-1), angiotensin II (Ang II), and COX-derived prostanoid and superoxide anions that prevent vasodilation [17]. In addition to the high risk of developing CVD, hypertension with age also increases the risk of other seemingly unrelated conditions, such as falls and fractures, which are caused by a decrease in cognitive and locomotor functions due to the development of vascular dementia and functional disability [20,21,22,23,24]. The study of new methods of treating hypertension, in particular age-related hypertension, is an important task for medical and pharmacological sciences. Animal modeling is of great benefit for understanding pathogenesis and therapy by employing experimental strategies that are not possible in human studies. There are several types of animal models of hypertension that aim to simulate hypertension by targeting an organ system. Models of hypertension currently used for preclinical studies are divided into the following types: genetic models, which include the genetically hypertensive rat, and the inherited stress-induced arterial hypertension rat model (SHR - animals with spontaneously developing hypertension in adulthood within 4 months [25,26]; the DSS rat—rats that are sensitive to a salt diet, in response to which they develop hypertension [27]; the fawn-hooded hypertensive (FHH) rat—rats with high blood pressure compared to Wistar rats [28,29]; Milan hypertension strain—rats that spontaneously develop hypertension immediately after weaning and plateau at 7–8 weeks of age [30,31]; the Lyon hypertensive rat—rats that spontaneously develop hypertension at the early age of 5 weeks [32,33]; and the Sabra hypertensive rat—rats sensitive to deoxycorticosterone acetate (DOCA) after unilateral nephrectomy [34,35]. Transgenic models of hypertension are largely aimed at studying the role of genes and their corresponding regulatory proteins in the pathogenesis and course of the disease. Transgenic models are divided based on the overexpression of a specific gene, for example, the mouse *Ren-2* gene and *TGR(mREN2*) [36]. Manifestations include marked cardiac hypertrophy, moderate proteinuria, and impaired endothelium-dependent relaxations [37,38]. For example, the effect of Angiotensin II in the proximal tubules was studied in angiotensin receptor (AT1A)–deficient mice. The expression of AT1A has been shown to be a necessary factor for the development of Ang II-mediated hypertension, as its deletion or overexpression in proximal tubules leads to hypotension or hypertension, respectively [39,40]. In rats and mice, the expression of human renin and angiotensin was studied by expressing the corresponding human genes in these animals [41,42], which helped not only determine the species-specificity of the RAAS, but also such transgenics are useful for investigating the function of different haplotypes and species-specific RAAS inhibition. There are transgenic mouse models for various syndromes, for example, Liddle syndrome [43], containing a gain-of-function stop codon in the β-epithelial sodium channel (reflecting the human mutation), in which animals develop hypertension on a high-salt diet. Gene knockdown represents a potential therapeutic strategy. For example, the administration of an adenovirus with direct anti-*miR* targeting the AT1A receptor to the paraventricular nucleus of the hypothalamus in SHR led to the suppression of hypertension [44]. However, species specificity in the genetic mechanisms of disease development should be taken into account [45]. The next type of hypertension models are experimentally induced hypertension models, such as the rhinovascular model—the first model of surgically induced hypertension. This model is based on mechanical clamping of the renal arteries, causing compression of the renal parenchyma, with resultant subtotal nephrectomy, resulting in decreased perfusion pressure and activating the RAAS, leading to vasoconstriction and salt and water retention, with systemic hypertension developing in a matter of days [46,47,48,49,50,51,52]. Pharmacological models, for example, long-term (4 weeks) subcutaneous administration of Ang II, which disrupts homeostasis by affecting the RAAS, causes increased blood pressure, myocardial hypertrophy, vascular remodeling, and chronic kidney disease, similar to that observed in people suffering from essential hypertension [53,54]. The inhibition of NOS by inhibitors such as L-NAME, leads to the development of endothelial dysfunction and increased blood pressure [55,56,57]. Despite the availability of a number of hypertension models described above, there is still a great need for a model that covers the maximum range of mechanisms involved in the development of primary hypertension [57]. Recently, animal studies in translational medical research have been increasingly questioned due to poor reproducibility, systematic errors, poor experimental design and poor quality of execution, analytical and logical errors and incomplete reporting [58,59,60,61,62]. The use of laboratory rodents as a test system for modeling hypertension has a number of advantages compared to the use of large animals. These advantages include the relatively low cost of animals and their short life span, which allows for lifelong or aging studies, and also allows the use of genetic changes (whole-body or cell-specific gene deletions (knockout) or gene editing) [57]. The most commonly used lines for preclinical studies performed in rats are the outbred stocks of Spraque Dawley and Wistar, which have been standard test systems for over 80 years [63,64]. According to the electronic library of scientific research (https://pubmed.ncbi.nlm.nih.gov/, assessed on 30 September 2024), 4700 articles were found when searching for Wistar rat, and 5535 articles for 2023 when searching for Spraque Dawley rat. Based on these data, it can be said that Wistar rats are the standard test system for preclinical studies and are used almost equally with Spraque Dawley rats. For these animals, a large pool of historical data on the toxicity of various substances has been accumulated. Wistar rats are much more frequently used than other strains in long-term 2-year carcinogenicity studies due to their stable lifespan, good social behavior (facilitates group housing, which reduces the cost of housing), low incidence of neoplastic changes with age in comparison with Spraque Dawley rats, and the presence of a large database on neoplastic changes accumulated over more than 25 years [65]. Preclinical toxicology studies, such as acute toxicity, chronic toxicity, carcinogenicity and reproductive toxicity, use animals of both sexes [63]. To model various pathological conditions, in particular those associated with the pathology of the cardiovascular system, such as myocardial infarctions and strokes, studies on the effects of various drugs on blood pressure, with or without hypertension modeling, are mainly carried out on males, since the hormonal cycles of females can influence blood pressure values [45,57,66,67,68,69,70,71]. It has previously been shown that hypertension can develop in Wistar rats with age [72]. Wistar rats are a standard test system for preclinical studies, used both in toxicological research and in studying the effectiveness of various drugs, including the use of various modeling methods. We propose to use age-related hypertension, which develops spontaneously in animals, as a physiological model for age-related hypertension to study new drugs for the treatment of hypertension. To achieve this, we measured blood pressure in animals of different ages—4, 10, 12 and 18 months. To divide animals as normotensive or hypertensive, we used the criterion of mean SBP, which for male Wistar rats is 115 mmHg [73]. We also decided to test the antihypertensive activity of the flavonoid-antioxidant taxifolin, which has previously established antihypertensive activity [74,75]. The purpose of our study was to experimentally test the hypothesis that Wistar rats develop arterial hypertension with age in a manner similar to humans, thereby creating a model of age-related hypertension in male Wistar rats, and also to establish the validity of this model by demonstrating the effectiveness of taxifolin in reducing high blood pressure.

## 2. Results

### 2.1. Development of Age-Related Hypertension (SBP Measurement Results)

**Figure 1:**
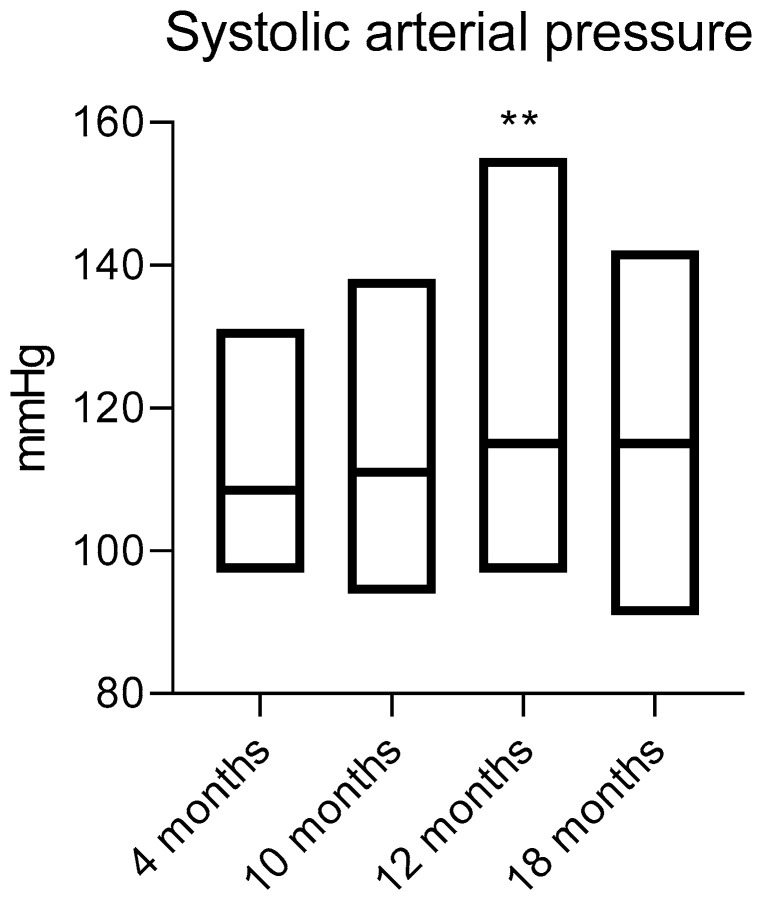
SBP for each age sample (4 months, 10 months, 12 months and 18 months) as a whole. ** *p* ≤ 0.01 relative to the 4-month age group with pairwise comparison Mann–Whitney U-test. Data are presented as median values (line at median).

When measuring SBP at the zero point in all animals of different ages, the average SBP value was as follows: for 4-month-old animals—110 ± 8 mmHg, for 10-month-old animals—114 ± 10 mmHg, for 12-month-old animals—118 ± 12 mmHg and for 18-month-old animals—115 ± 11 mmHg. The SBP of 12-month-old animals was statistically significantly higher than that of 4-month-old animals (Figure 1).

**Figure 2:**
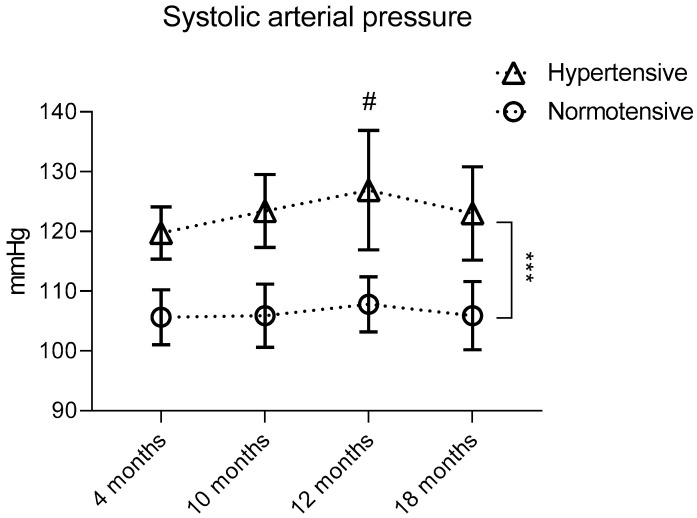
Initial SBP when divided into groups—normotensive (SBP less than or equal to 115 mmHg) and hypertensive (SBP above 115 mmHg)—within each age (4, 10, 12 and 18 months). *** *p* ≤ 0.001 relative to groups with normal blood pressure within each age with pairwise comparison using the Mann–Whitney U-test, # *p* ≤ 0.05 relative to animals aged 4 months with pairwise comparison using the Mann–Whitney U-test.

After measuring the SBP in all animals, they were divided into two groups: normotensive, with the SBP below 115 mmHg, and hypertensive, with the SBP equal to or above 115 mmHg. This division was carried out among animals of each age, and the SBP value in normotensive animals was statistically significantly lower than the SBP values in hypertensive animals (106 ± 4 mmHg versus 120 ± 4 mmHg in 4-month-old animals, 106 ± 5 mmHg versus 123 ± 6 mmHg in 10-month-old animals and 108 ± 5 mmHg versus 123 ± 8 mmHg in 18-month-old animals). Thus, in male Wistar rats, starting from 10 months of age, age-related hypertension develops, and in 12-month-old hypertensive animals, the SBP value is significantly higher compared to hypertensive 4-month-old animals (Figure 2).

**Figure 3:**
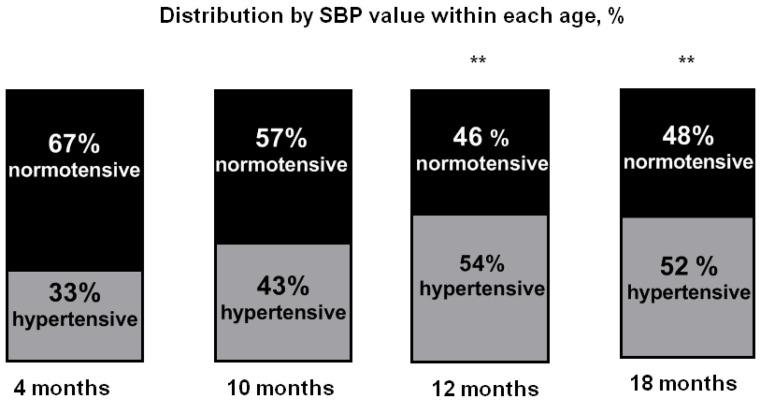
Percentage ratio of normotensive and hypertensive animals within the sample of each age (4 months *n* = 42, 10 months *n* = 30, 12 months *n* = 41 and 18 months *n* = 42). ** *p* ≤ 0.01 relative to animals aged 4 months according to the Chi square test.

Although hypertensive animals were present across all ages, their percentage varied depending on age. Hypertensive animals made up 33% (14 out of 42 animals) of the total number among 4-month-old animals, 43% (13 out of 30 animals) among 10-month-old animals, 54% (22 out of 41 animals) among 12-month-old animals and 52% (22 of 42 animals) among 18-month-old animals. Based on the data obtained, we can conclude that the percentage of hypertensive animals increases with age, which, from 12 months onwards, is statistically higher than that in 4-month-old animals (Figure 3).

Among the animals whose SBP was equal to or exceeded 115 mmHg, a cohort of animals was identified whose SBP was 10 mmHg higher, that is, it was greater than or equal to 125 mmHg (Table 1).

It has been shown that with increasing age there is an increase in the absolute and relative number of animals whose SBP exceeds 125 mmHg.

The mean values of SBP differed significantly across all ages between the cohorts of hypertensive animals with blood pressures of 115 mmHg, 124 mmHg and greater than or equal to 125 mmHg (Table 2).

### 2.2. Effect of Taxifolin on SBP

Since the number of animals with blood pressures above 125 mmHg in the groups of 4- and 10-month-old animals was too small to form groups, it was not possible to consider them as separate groups to study the effect of the substance taxifolin on the value of the SBP. Therefore, the hypotensive effect of taxifolin was studied in groups of hypertensive animals whose SBP was greater than or equal to 115 mmHg.

**Figure 4:**
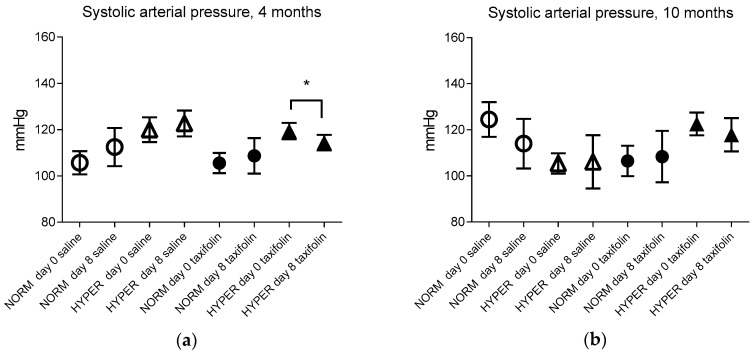

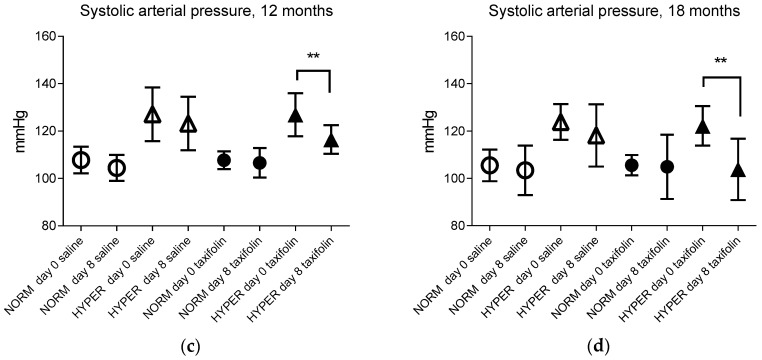
The effect of taxifolin on the SBP of animals of each age (**a**) 4 months, (**b**) 10 months, (**c**) 12 months, (**d**) 18 months. NORM—normotensive animals (initial SBP is less than or equal to 115 mmHg). HYPER—hypertensive animals (initial SBP is above 115 mmHg). * *p* ≤ 0.05, ** *p* ≤ 0.01 relative to day 0 in each group (normotensive or hypertensive) with pairwise comparison using the Mann–Whitney U-test.

A weekly course of taxifolin administration led to a pronounced decrease in the SBP in hypertensive animals of all ages (not significant in 10-month-old animals), but did not affect the value of the SBP in normotensive animals (Figure 4).

## 3. Discussion

We have shown that in a significantly large sample of male Wistar rats of each age, namely 4 months, 10 months, 12 months and 18 months, the average SBP does not fall outside the normal SBP range (115 mmHg) [73], differing from it insignificantly. However, when applying cluster analysis using the parameter values of SBP ≥ 115 mmHg and SBP < 115 mmHg, we identified groups of animals in the same sample that differed significantly in SBP values. It has been shown that the number of hypertensive animals increases with age. In addition, within the cluster of animals with an SBP < 115 mmHg, two cohorts of animals with an SBP up to 125 mmHg and with an SBP above 125 mmHg were identified. It has been shown that the number of animals with an SBP above 125 mmHg increases with the age of the animals. In summary, this study shows that male Wistar rats develop hypertension over the course of their lives, which worsens with age. Wistar rats are used as test systems in preclinical studies without categorizing them by their SBP value, but, as we have shown, the specific effect of drugs may be different in these animals when categorized by their SBP value. In our case, we demonstrated that taxifolin affects SBP only in hypertensive animals. When creating genetic models of hypertension (without interfering with the genome), animals are selected for high blood pressure in order to further crossbreed and produce animals with elevated SBP [25,27,28]. However, we propose to use our model of age-related hypertension using the general animal population routinely used in preclinical studies, dividing them into hypertensive and normotensive animals during the study (after measuring their SBP). This model is physiological and is as close as possible to the development of primary hypertension in the human population. The mechanisms of development of age-related hypertension are actively studied by modern science, and include the mutual influence of pathological factors, such as chronic inflammation, oxidative stress and vascular dysfunction. These factors create a vicious cycle, and contribute to the aggravation of pathology [15,16,17]. To demonstrate the hypotensive effect, we chose the drug taxifolin based on previous studies in which it was shown that taxifolin reduces SBP in hypertensive-aged male Wistar rats [72]. The effect of reducing SBP after taxifolin therapy is most likely due to the relaxation of the endothelium due to ACE inhibition and antioxidant action, as was shown in the study by Arutyunyan et al., 2018 [74]. The study revealed a decrease in the activity of angiotensin-converting enzyme in the aorta of old rats receiving 100 μg/kg of taxifolin for 5–12 days. This work also showed that the administration of taxifolin at a dose of 100 μg/kg while taking the NO inhibitor LNAME in 11-week-old rats leads to a decrease in the level of reactive oxygen and nitrogen species in the aorta. Also, the hypotensive effect of taxifolin, due to its effect on ACE activity, was shown in the work of Korystova et al. 2018, where a 7-day administration of taxifolin against the background of NOS inhibition normalized ACE levels and reduced SBP [75]. Moreover, it was previously shown that intragastric administration of taxifolin at a dose of 100 mg/kg and 300 μg/kg for 2 weeks has no effect on the SBP of SHR rats [76]. Also, in the work of Plotnikov et al. 2017 it was shown that intragastric administration of taxifolin at a dose of 50 mg/kg during the period of development of hypertension in SHR, namely from the 6th to 12th week of life, did not affect the formation of age-mediated hypertension in animals, although it improved local cerebral blood flow in the visual cortex of SHR [77]. In a study by Jasenovec et al. 2022, the administration of taxifolin within a drink at a dose of 20 mg/kg/day for 14 days (the age of the animals was not specified) led to a decrease in SBP in SHR. However, this decrease was not close to normalization of blood pressure, and the values after blood pressure reduction were at a high level of hypertension (156.8 ± 9.9 mmHg) [78]. In a study by Liskova et al. 2023, oral administration of taxifolin at a dose of 20 mg/kg for 10 days in SHR rats led to a decrease in SBP by 10 units at an initial blood pressure of 173 ± 3 mmHg, which also cannot be considered a decrease to the physiological norm [79]. At the same time, the significant effectiveness of the hypotensive effect of taxifolin was demonstrated in our presented physiological model of hypertension, which develops over the lifespan and is not caused by special selection and crossbreeding for hypertensiveness, in contrast to genetically determined hypertension, as is the case with SHR rats. We can conclude that although Wistar rats develop and experience worsening hypertension with age, these animals are not prone to developing severe hypertension unlike SHR rats. This model of age-related hypertension can be classified as a model of high blood pressure/stage I hypertensive disease.

## 4. Materials and Methods

### 4.1. Animals

The study used sexually mature Wistar males of different ages, namely 4-month—42 individuals, 10-months—30 individuals, 12-months—41 individuals and 18-months—42 individuals. Animals were obtained from the Pushchino Laboratory Animal Nursery, Pushchino, Russia. All animals were of SPF status. All procedures and manipulations with animals were approved by the Committee for Control over Care and Use of Laboratory Animals of BIBCh RAS (IACUC) (protocol number 780/21 from 14/12/2021) and were carried out in accordance with the EU Directive 2010/63 /EU. After being received from the nursery, the animals underwent adaptation within 7 days. During this period, the animals were monitored for signs of deviation in health status. Animals without signs of health deviations were selected for the experiment (clinical examinations were conducted). During the study, the animals were kept under controlled environmental conditions in a barrier zone with a “clean” and “dirty” corridor system with controlled environmental conditions, including a temperature of 20–24 °C, relative humidity of 30–55% and a 12-h light cycle (08:00–20:00—”day”, 20:00–08:00—”night”), with a 10-fold change in air volume in the room per hour. The animals received a complete autoclaved food for laboratory mice and rats, Velaz FORTI 1324 Maintenance Diet (Altromin Spezialfutter GmbH & Co KG, Im Seelenkamp 20, D-32791 Lage, Germany) ad libitum.

### 4.2. Design Description

**Figure 5:**
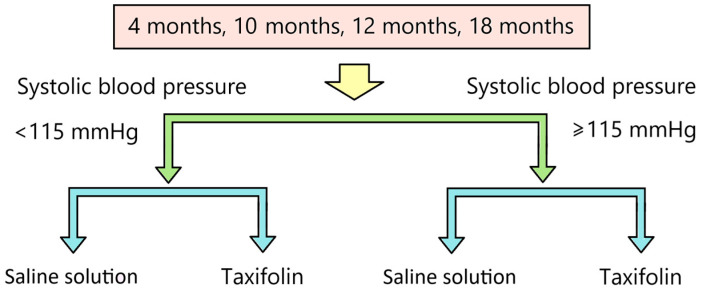
Scheme of distribution of animals in the experiment according to the value of SBP and administration of taxifolin.

SBP was measured in all animals; based on the results of this measurement, animals were divided into subgroups within each age. Animals were divided into normotensive—those whose SBP was less than 115 mmHg, and hypertensive—those whose SBP was equal to or above 115 mmHg. After being divided into normotensive and hypertensive animals, they were randomly divided within these subgroups into two more subgroups (Figure 5). One of the subgroups received taxifolin at a dose of 10 µg/kg intraperitoneally for 7 days, and the second received saline. The volume of administration was 10 mL/kg. At the end of the administration, the animals’ SBP was measured again.

### 4.3. Measuring SBP Using a Tail Cuff

SBP measurements were performed non-invasively using a computerized system PowerLab 8/35 ADInstruments Pty Ltd., Bella Vista, Australia, with a tail cuff sensor Pulse Transducer/Pressure Cuff for NIBP manufactured by Panlab Harvard Apparatus, Barcelona, Spain for ADInstruments Pty Ltd.

**Figure 6:**
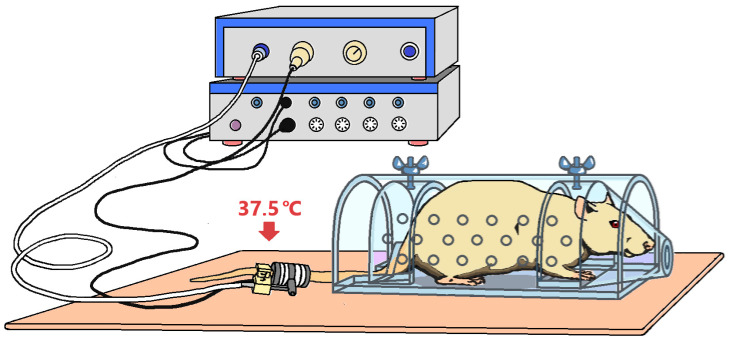
The process of measuring SBP using a tail cuff in rats.

**Figure 7:**
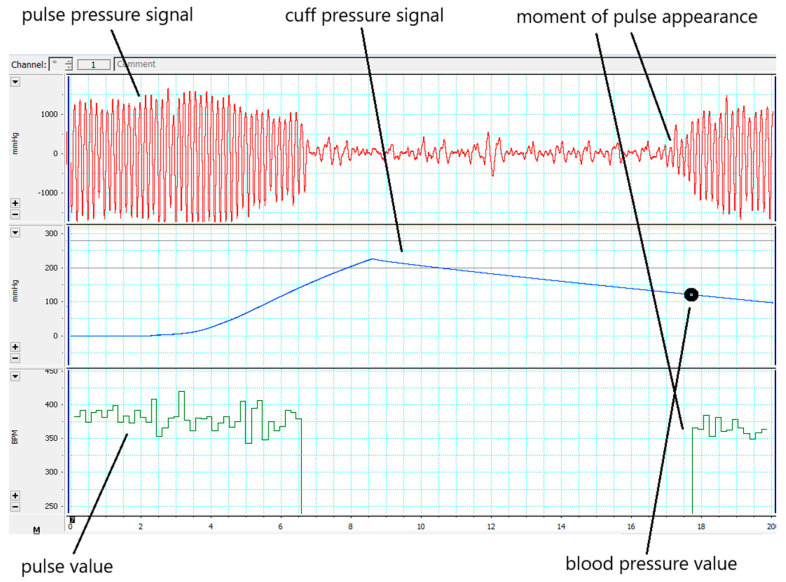
Screenshot from the PowerLab screen when measuring SBP using a tail cuff.

The animal was placed in a holding house of a suitable size, which was located on a thermostatically controlled substrate (28–30 °C) to improve blood circulation in the tail. The cuff sensor was positioned at the base of the animal’s tail so that the clip with the sensor was located on the ventral surface of the tail directly below the caudal artery (Figure 6). After the animal adapted to the holding house, the cuff compressor was activated and the appearance of a pulse wave was recorded, which disappeared after the tail vein was clamped with the cuff. The pressure in the cuff at which the pulse wave appeared corresponded to the systolic blood pressure in the animal’s tail artery (Figure 7).

### 4.4. Administration of Taxifolin/Vehicle

The taxifolin solution was prepared by dissolving a sample of taxifolin powder (Sigma, Supelco, Bellefonte, PA, USA) in saline at a concentration of 10 µg/mL. The administration of taxifolin at a dose of 100 µg/kg or a carrier–saline solution, was carried out intraperitoneally once a day for 7 days. The volume of administration was 10 mL/kg.

### 4.5. Statistical Evaluation

For all data, average values and standard deviations were calculated using Microsoft Excel. Comparisons between groups were performed using the Statistica for Windows v.5 program using the Mann–Whitney U-test for pairwise comparisons. Intergroup differences were considered significant when p ≤ 0.05.

## 5. Conclusions

Throughout life, starting from 10 months, male Wistar rats develop age-related hypertension, which worsens with age, and is corrected by a drug with hypotensive activity, known as taxifolin. This condition can be used as a physiological model of age-related hypertension in male Wistar rats to study the effects of antihypertensive drugs. It is possible to use hypertensive Wistar males of any age to study the characteristics of therapeutic and toxicological effects on the SBP. In addition, possible differences in drug effects between normotensive and hypertensive animal cohorts should be considered when conducting nonclinical toxicology studies.

## Figures and Tables

**Table 1:** Absolute and relative numbers of hypertensive animals in cohorts with SBP below and above 125 mmHg.

Animal Age	Number of Animals		Percentage of Animals with SBP Greater than or Equal to 125 mmHg
	SBP from 115 to 124 mmHg	SBP from 125 mmHg and Above	
4 months	12	2	14%
10 months	8	5	38%
12 months	10	12 *	55%
18 months	13	10	43%

* *p* ≤ 0.05 relative to 4-month-old animals according to the Chi-square test.

**Table 2:** SBP of hypertensive animals, divided into cohorts according to SBP values below and above 125 mmHg.

Animal Age	SBP from 115 to 124 mmHg	*n*	SBP from 125 mmHg and Above	*n*
4 months	118 ± 2	14	129 ± 4 *	2
10 months	120 ± 3	8	129 ± 5 **	5
12 months	119 ± 3	10	134 ± 8 ***	12
18 months	117 ± 2	13	131 ± 5 ***	10

* *p* ≤ 0.05, ** *p* ≤ 0.01, *** *p* ≤ 0.001 relative to the group SBP 115 – ≤ 125 mmHg according to the Mann–Whitney U-test in pairwise comparison.

## Data Availability

All individual data for all animals can be obtained upon request from the corresponding author.
